# A New Classification and Clinical Predictivity for Some Naevus Variants

**DOI:** 10.5402/2011/536752

**Published:** 2011-07-14

**Authors:** G. Fabbrocini, C. Mazzella, F. Pastore, A. Monfrecola, M. C. Annunziata, M. C. Mauriello, V. D'Arco, C. Marasca, V. De Vita

**Affiliations:** Division of Clinical Dermatology, Department of Systematic Pathology, University of Naples “Federico II”, Via Sergio Pansini 5, 80133 Napoli, Italy

## Abstract

*Background*. The incidence of cutaneous melanoma is rapidly increasing in Europe. Active research is directed toward the identification of naevi as a risk factor. *Objective*. The aim of our case-control study was to observe different numbers of moles and different mole typology associations in order to evaluate clinical predictivity and to establish a new classification for some naevus variants. *Methods*. A case-control study was carried out, enrolling 64 cases affected by melanoma and 183 controls, between October 2009 and February 2011. Each patient was interviewed and subjected to clinical examination. The resulting data were analysed using the statistical elaboration program SPSS 16.0. *Results*. The association of target naevus with other variants increases the degree of risk (target + small brown Odds Ratio 5.25; confidence interval 1.8–15.4); (target + small brown + small black + large brown odds ratio 5.0; confidence interval 1.1–22.4). Therefore, other variants and/or other variant combinations do not significantly increase risk. *Conclusion*. People presenting two naevus variants in association with other naevus variants seem to run a major risk. The general nonuniformity of the whole naevus panorama should be carefully considered.

## 1. Introductions

The incidence of cutaneous melanoma (CM) is rapidly increasing in Europe, especially in the Caucasian population [1]. Incidences are known to vary amongst white populations, reaching 15 cases per 100,000 inhabitants per year in Scandinavia and 5–7 cases per 100,000 inhabitants per year in Mediterranean countries [2–4].

Fortunately, these data are matched by active research directed toward a greater identification of risk factors linked with the onset of melanoma. In this regard, the presence of naevi assumes particular importance as an independent and very high risk factor for the development of melanoma. Clinically dysplastic naevi and an excess number of melanocytic naevi are among the most serious known risk factors for cutaneous melanoma.

In the last decade, a plethora of investigations aimed at identifying risk factors for the development of melanoma have been conducted. More recently, working on 200 patients affected by melanoma and 200 healthy control patients, Nikolaou et al. confirmed that the presence of atypical naevi is an important risk factor for the development of CM, tripling the odds ratio (OR) [9]. According to Carli et al. [10], the greatest difference between subjects with or without atypical naevi is related to their number of common naevi: the authors demonstrated that more than 30 naevi are encountered in 41.5% of melanoma cases, but in only 9% of healthy control subjects (OR 8.0; Confidence Interval (CI) 6.3–10.3).

Even if atypical clinical features of naevi constitute a very high risk factor for CM, their partial correspondence with histologic dysplasia [11] indicates that the total number of naevi remains the most important factor in the development of CM. These data confirm the major risk of dysplasia or the development of CM when the total number of naevi exceeds 100 [12], and the presence of an elevated number of naevi needs to be carefully evaluated in the panorama of risk factors for the development of melanoma [13, 14]. 

As a result, the clinical and physiopathological study of naevi is acquiring ever greater importance. Numerous studies have been undertaken in order to establish a classification of naevi and to define their physiological and pathological evolution, the majority of which have aimed to correlate the macroscopic appearance of these structures with their histopathological characteristics [15, 16], dividing them into dermal, composed, and junctional [17]. 

Kincannon and Boutzale further divided acquired naevi into five categories according to their rough macroscopic appearance and their relationship to the skin surface, noticing that the papillomatous variant is most commonly associated with such phenotypes as blue or green eye colour and fair, light brown, or red hair. These authors underlined the importance of any possible correlations between naevi and phenotype characters and also drew up a more accurate and detailed classification of naevi, even though it was restricted almost exclusively to dermal naevi [18]. 

Gallagher and Kwan showed that naevi have a preferred body distribution in the two genders which is already evident in the pediatric age: in males naevi are more numerous on the head, neck, and trunk, whereas females show a greater distribution on their limbs. Naevi are much more numerous in children intermittently exposed to the sun, highlighting the importance of sun exposure during childhood in the genesis of benign acquired naevi. However, the same distribution pattern in photoprotected locations raises the possibility that hormonal differences play a consistent role in the development of melanocytic naevi [19, 20].

The most famous and widely accepted classification of acquired melanocytic naevi was published by Ackermann and Magana-Garcia who classify naevi into four types, of which Clark naevi constitute the predominant type among commonly acquired melanocytic naevi. When their diameter exceeds 5 mm and at least two other signs of clinical activity are present, such as irregular or poorly defined edges, uneven colouring, or erythematous patch variations in skin pattern, they are defined as atypical, whereas when histology shows the presence of specific architectural features they are diagnosed as dysplastic naevi [21].

However, although Ackerman's classification continues to be a reference, all pigmented melanocytic naevi are grouped under the single denomination of Clark naevi which does not enable the analytical investigation of naevi in the field of melanoma prevention: further steps in this direction have only led to the conviction that the presence of a small number of Clark naevi has weak meaning in young patients with no melanoma familiarity. On the other hand, when Clark naevi arise in older subjects, between the ages of 40 and 50 with no familiarity, their meaning is less clear but it is considered as an anomaly of the normal formation and growth process of melanocytes [5]. 

Several variants, whose denominations can be found in the literature, are mentioned and defined by means of clinical criteria in Saurat's book [6]. This publication includes the most common naevi present in the population; Sutton or Halo naevus is also mentioned and congenital pigmented naevi are divided into small (<1.5 cm), medium (1.5–20 cm), and large (>20 cm). Further progress in this direction can be achieved by making a precise systematic table of the various subspecies of naevus, free of any excess or overlapping.

## 2. Aims

We have conducted this case-control study with the aim of observing different numbers of moles and different mole typology associations between patients affected by melanoma and control volunteers. The analysis of naevus variants is not only of commercial value but can be of considerable practical value in the self-analysis of naevi, which is of great importance in the prevention of CM. This analysis makes it easier for patient and physician to know the number and distribution of the naevi, thus enabling the timely observation of any anomalous cellular activity in progress, of any lesions at risk or of a still undetected early clinical melanoma. In addition, this study may provide a further contribution to the determination of a naevic map in the patient undergoing a routine dermatological checkup without suspect lesions, evaluating the possibility of identifying one or more categories of naevus variants for every individual in order to identify further guide lines for the study of melanoma risk factors. 

Such classifications can upgrade the clinical phase of mole analysis. Naevus variants can improve the interaction between doctors and their patients, who will be able to furnish more accurate information on the naevi and their evolution, thus providing doctors with better “guidelines” for diagnosis and therapy. In this way, they will be able to better orient themselves in the identification of risk naevus/naevi, examining mole variant associations and their numerosity, according to the total number of naevi and the phenotypic features of the people concerned. One of the aims our study is to try to identify the phenotypic features of individual naevus variants with a view to establishing different risk patterns for MC, which may in turn suggest different follow-up procedures.

## 3. Material and Methods

A case control study was carried out. 64 cases and 183 controls were selected amongst patients at the Dermatology Department of Federico II University, Naples between October 2009 and February 2011. The cases and the controls (102 males and 145 females) had an average age of 34.8 years (minimum 2 and maximum 82). The 64 cases were equally distributed between the two sexes; of the 183 controls 70 were males and 113 females. The cases enrolled were patients affected with nodular or superficial melanoma, the thickness of the lesion had been determined in each case (staging model AJCC 1988), and the time elapsed from the excision was calculated as between 0 and 10 years. Patients with melanoma that had developed on a malignant freckle were excluded, as these melanomas rarely break out in association with a preexisting naevus. The controls were first-degree relatives of cases living in the same household for the same period. All patients were Caucasian and homogeneous in respect of socioeconomic indicators and occupation (Table 2).

### 3.1. Interviews

Before the naevus count, a trained interviewer compiled a form for each case and control. The interview lasted about 15 minutes and included the following data: residence, phenotype characteristics such as eye colour (brown, hazel, green, and blue), hair colour (black, brown, fair, and red); phototypes for groups and subgroups from I to IV, (since V and VI are practically absent in our area for ethnic reasons), melanoma familiarity, sunburn history subdivided by age (juvenile 0–15 years old, and adult over 15 years old), and use of sun beds.

### 3.2. Naevus Count

The naevi were evaluated after being classified into one of three major groups: the common acquired melanocytic naevi (NMAC) (>2 mm), congenital naevi, and atypical naevi [22].

NMAC presence was evaluated on the entire body surface. The location of each one was reported: head or neck, arm, upper arm, hands, chest, abdomen, upper back, lower back, thigh, and feet. Sun freckles or ephelides were recorded by a number code (l = none, 2 = from 1 to 5, and 3 = more than 5). 

Those naevi which were identifiable as atypical were reported separately from the total count.

In order to minimise possible errors in the naevus count and according to data reported in the literature, a series of clinical criteria identifying the variants was established (Table 1), based on clinical morphological criteria, that considered the following:

- dimension,
- colour,
- relationship to the skin surface,
- shape.

This was used by the dermatologists to identify every naevus lesion.

For each variant, the presence of naevic elements and their number was reported, as well as the total number of variants present and the total number of naevi >2 mm, regardless of their variant group membership.

### 3.3. Data Analysis

The data were analysed using the statistical elaboration program SPSS 8.0. Univariate analysis was carried out, followed by a chi-square test to evaluate the degree of association between several variables. Finally, a multivariate analysis of stepwise logistic regression was performed. 

The OR and the 95% confidence limit were adjusted for age, gender, number of naevi, and some known risk factors for CM, such as phototype I-II, melanoma familiarity, and past sunburn history.

## 4. Results

The sample was found to be homogeneous for the most important known factors. More than 70% of the sample had homogeneous phenotypes: phototype II or III; dark hair; dark eyes (9% of the subjects phototype I, 40% phototype II; 35% phototype III; 14% phototype IV). The sample was also homogeneous in relation to the total number of naevi: 42% of cases and 45% of controls, thus nearly half the sample reported a total number of naevi ranging between 10 and 50 elements. 

67% of cases and 80% of controls were negative for childhood sunburn: more than 75% of the sample did not present a childhood sunburn risk factor.

With regard to our first aim, that is, the distribution of the single types in the sample, our study showed that the small brown naevus was present in more than 70% of controls and of cases. The raised brown naevus, on the other hand, was found in 93% of control subjects and in only 7% of cases. The target naevus was identified in 7.8% of the cases and in 16.1% of the controls.

The association small brown + small black is equally distributed between the two groups: 27% of the cases and the controls.

The presence of the target naevus varies in the two groups when it is the only type present and, when in association with others, more predominantly in cases (target: 16% of cases and 8% of controls; target + small brown: 16% of cases and 7% of controls; target + small brown + small black: 10% of cases and 3% of controls; target + small brown + small black + large brown: 8% of cases and 2% of controls).

The presence of the small brown does not seem to be associated with CM (small brown OR 2.05; CI 1.0–4.42). The risk increases when there is also the target variant (target OR 3.65; CI 1.4–9.7). The raised brown naevus, on the other hand can be considered as protective for the development of CM (raised nevus OR 0.2; CI 0.03–0.8).

It can be seen that among subjects with an equal number of naevi, a greater number of variants corresponds to a greater cutaneous melanoma risk. The results were relative to the variants indicated in Table 3. With an equal number of naevi, the combination of only two variants was found to be of slight significance: (small black + large brown OR 3.5; CI 1.3–9.2); (target + large brown OR 3.6; CI 1.1–11.2); (small black + large brown + large black OR 5.5; CI 1.4–21.7); (target + small brown + small black OR 5.9; CI 1.5–23.2) indicating that apart from the number of naevi, naevus variants might influence the degree of risk.

On the other hand the OR does not change when the small brown variant is added to the association of small black and small brown (small brown + large brown + small black (OR 3.5; IC 1.3–9.5), small black + large brown (OR 3.5; IC 1.3–9.2)).

Analysing these data, it can be seen that a further significant element is provided by the specific impact of the different variants: the data indicate that the presence of the very common small brown naevus, does not modify the OR, whereas the target naevus requires more careful evaluation (OR 3.65; CI 1.4–9.7).

Moreover, the association of the target naevus with other variants, increases the degree of risk [target + small brown OR 5.25; CI 1.8–15.4]; [target + small brown + small black OR 5.9; IC 1.5–23.2]; [target + small brown + small black + large brown OR 5.0; IC 1.1–22.4].

Our study indicates that some variants and/or variant combinations, do not significantly increase risk. Miescher naevus; Unna naevus and fried egg naevus are completely devoid of meaning. (fried egg OR 0.4; CI 0.08–1.86; *P* = 0.23); (Unna OR 0.94; CI 0.46–1.90; *P* = 0.8); (Meischer OR 1.26; CI 0.5–2.9; *P* = 0.6).

The presence of the raised brown naevus actually acts as a protective factor.

## 5. Conclusion

The self-assessment of pigmented lesions remains an important step in the prevention of skin melanoma. In an Australian study, carried out to determine the correspondence between the patient's and the dermatologist's naevus check, It was shown that the number of pigmented lesions is always underestimated by the patient, but agreement increases for atypical melanocytic naevi [22]; however, the role of the atypical naevus has been the subject of much debate in the literature and elsewhere, and, of course, it is not so easy to diagnose clinically.

Our study aims to introduce the differentiation of naevus variants among the monitoring criteria of risk factors for cutaneous melanoma. The results show that even if the total number of naevi represents a risk factor for the development of cutaneous melanoma, orientation in the naevus panorama of a specific subject requires the identification of several naevus variants. The most common naevus variants, such as dermal naevi and homogeneously pigmented naevi (small and large brown or small and large black naevi) cannot alone represent a risk marker; however the combination of two or more variants becomes a significant risk marker, and their combination in a person thus enables the identification of groups of subjects as reference targets potentially at risk of developing cutaneous melanoma.

On the other hand, people presenting the target naevus in association with other naevus variants, have a major rearrangement and activity of pigmented lesions, and therefore require a more rigorous follow-up procedure. The greater variability of this variant is perhaps also expressed through its greater clinical inhomogeneity. This introduces a further aspect of our results: it is important to consider carefully not only the non-uniformity of a single type but also the general non-uniformity of the whole naevus panorama. The data actually available in the literature indicate that when the total number of naevi present in a subject increases, the risk of developing cutaneous melanoma also increases. A further datum emerges from our study: when two subjects present a similar number of naevi, the greater risk for cutaneous melanoma is linked to the greater number of naevus variants. This datum is clinically substantiated in the differentiation of the monitoring of pigmented lesions. Subjects having a moderate number of lesions but presenting clinical inhomogeneous pigmentation are included in the group of subjects at risk. Conversely, subjects with an elevated number of naevi but with a homogeneous panorama and low variability, can be observed with greater tranquillity, without however neglecting other risk factors.

The classification of naevus in variants can be an indicator of clinical predictivity particularly in subjects who present naevi and especially an elevated number of naevi. Furthermore, some variants considered singularly can play a key role in determining the clinical picture of the subject and are to be evaluated with great attention. Consider, for example, naevi such as the small brown naevus whose presence or absence appears to be totally uninfluential, whereas the presence of others, such as the bullseye naevus, can greatly modify the dermatologist's attitude toward the patient.

## Figures and Tables

**Table 1:** Naevus variants following the literature's classification^∗^.

Naevus variant	Dimension	Colour	Shape	Relationship to skin surface
Small brown naevus [5]	<5 mm	Brown	Roundish	Flat
Small black naevus [5]	<5 mm	Black	Roundish	Flat
Large brown naevus [5]	>5 mm	Brown	Roundish/oval	Flat
Large black naevus [5]	>5 mm	Black	Roundish/oval	Flat
“Fried egg” naevus [5]	—	Varies from brown to black	Variable	Central part raised with respect to the periphery
Target naevus [6]	—	Varies from light to dark brown	Roundish/ovai	Flat
Raised brown naevus [7]	—	Pinkish-brown	Roundish	Raised with smooth surface
Moriform Unna's naevus [8]	—	Varies from light brown to pinkish brown	Blackberry	Raised
Dome-shaped Miescher's naevus [8]	—	Brown	Dome shaped	Raised
Sutton's patch naevus [8]	—	Achromatic peripheral patch	Roundish oval elliptic	Flat
Spitz'pink naevus [8]	—	Pink-beige	Modular	Raised

^∗^Our study is based on naevi with a diameter greater than 2 mm.

**Table 2:** Distribution of the 64 cases and 183 controls for the University Federico II of Naples.

Features	Case	Control
Sex		
Male	32	70
Female	32	113

Age		
<35	16	122
36–45	11	27
46–55	14	18
56–65	11	6
>65	10	7

Phototype		
I	7	17
II	33	67
III	17	69
IV	7	28

Sunburn during childhood		
No	42	147
Yes	22	34

Sunburn during old age		
No	41	150
Yes	23	31

Small brown		
No	16	55
Yes	46	125

Small black		
No	33	108
Yes	29	72

Large brown		
No	41	116
Yes	21	64

Large black		
No	55	163
Yes	7	17

Target		
No	52	166
Yes	10	14

Fried egg		
No	60	162
Yes	2	18

Raised brown		
No	60	153
Yes	2	27

Unna		
No	46	123
Yes	16	57

Miescher		
No	51	155
Yes	11	25

Small brown + small black		
No	45	132
Yes	17	48

Small black + small brown + large brown		
No	50	158
Yes	12	22

Small brown + small black + large Brown + large black		
No	57	175
Yes	5	5

Small brown + small black + large brown + large black + target		
No	61	178
Yes	1	2

Small black + large brown		
No	49	156
Yes	13	24

Small black + large brown + large Black		
No	57	175
Yes	5	5

Small black + large brown + large black + target		
No	61	178
Yes	1	2

Large brown + large black		
No	57	172
Yes	5	8

Large brown + large black + target		
No	61	177
Yes	1	3

Large black + target		
No	61	177
Yes	1	3

Large brown + small brown		
No	42	121
Yes	20	59

Large black + small brown		
No	55	167
Yes	7	13

Large black + small brown + small black		
No	56	171
Yes	6	9

Large black + small brown + small black + target		
No	61	178
Yes	1	2

Large black + small black		
No	56	168
Yes	6	12

Target + small brown		
No	52	168
Yes	10	12

Target + small brown + small black		
No	56	175
Yes	6	5

Target + small brown + small black + large brown		
No	57	176
Yes	5	4

Target + small black		
No	56	173
Yes	6	3

Target + large brown		
No	54	169
Yes	8	11

Target + large black		
No	61	177
Yes	1	3

**Table 3:** Odds ratio (OR) and 95% confidence interval (CI) of develop of cutaneous melanoma in relation to the presence of one naevus variants or their association.

	Case/controls	OR^a^ (95% CI)
Small brown		
No	16/55	1.0
Yes	46/125	2.05 (0.95–4.42)

Small black		
No	33/108	1.0
Yes	29/72	1.46 (0.76–2.78)

Large brown		
No	41/116	1.0
Yes	21/64	1.65 (0.81–3.37)

Large black		
No	55/163	1.0
Yes	7/17	1.42 (0.5–4.05)

Target		
No	52/166	1.0
Yes	10/14	3.65 (1.38–9.65)

Fried egg		
No	60/162	1.0
Yes	2/18	0.4 (0.08–1.86)

Raised brown		
No	60/153	1.0
Yes	2/27	0.17 (0.03–0.84)

Unrta		
No	46/123	1.0
Yes	16/57	0.94 (0.46–1.90)

Miescher		
No	51/155	1.0
Yes	11/25	1.26 (0.5–2.9)

Small brown + small black		
No	45/132	1.0
Yes	17/48	1.91 (0.78–4.67)

Small black + small brown + large Brown		
No	50/158	1.0
Yes	12/22	3.55 (1.33–9.48)

Small brown + small black + large brown + large black		
No	57/175	1.0
Yes	5/5	0.18 (0.04–0.72)

Small brown + small black + large brown + large black + target		
No	61/178	1.0
Yes	1/2	2.5 (0.21–30.6)

Small black + large brown		
No	49/156	1.0
Yes	13/24	3.5 (1.32–9.24)

Small black + large brown + large black		
No	57/175	1.0
Yes	5/5	5.48 (1.38–21.72)

Large brown + large black		
No	57/172	1.0
Yes	5/8	2.65 (0.71–9.82)

Large brown + large black + target		
No	61/177	1.0
Yes	1/3	0.67 (0.10–17.56)

Large brown + small brown		
No	42/121	1.0
Yes	20/59	2.40 (0.9–6.05)

Large black + small brown		
No	55/167	1.0
Yes	7/13	1.8 (0.57–5.65)

Large black + small brown + small black		
No	56/171	1.0
Yes	6/9	2.82 (0.82–9.71)

Large black + small black		
No	56/168	1.0
Yes	6/12	2.16 (0.67–6.95)

Target + small brown		
No	52/168	1.0
Yes	10/12	5.25 (1.78–15.4)

Target + small brown + small black		
No	56/175	1.0
Yes	6/5	5.89 (1.5–23.16)

Target + small brown + small black + large brown		
No	57/176	1.0
Yes	5/4	4.9 (1.1–22.4)

Target + small black		
No	56/173	1.0
Yes	6/7	4.01 (1.12–14.4)

Target + large brown		
No	54/169	1.0
Yes	8/11	3.6 (1.14–11.27)

Target + large black		
No	61/177	1.0
Yes	1/3	1.66 (0.15–17.5)

^a^Adjusted by age, gender, and number of naevi.
